# High glucose-induced STING activation inhibits diabetic wound healing through promoting M1 polarization of macrophages

**DOI:** 10.1038/s41420-023-01425-x

**Published:** 2023-04-26

**Authors:** Kang Geng, Xiumei Ma, Zongzhe Jiang, Wei Huang, Junling Gu, Peng Wang, Lifang Luo, Youhua Xu, Yong Xu

**Affiliations:** 1 Faculty of Chinese Medicine, State Key Laboratory of Quality Research in Chinese Medicine, Macau University of Science and Technology, Avenida Wai Long, Taipa Macao; 2 Department of Endocrinology and Metabolism, Metabolic Vascular Disease Key Laboratory of Sichuan Province, Sichuan Clinical Research Center for Nephropathy, Cardiovascular and Metabolic Diseases Key Laboratory of Luzhou, Luzhou, Sichuan PR China; 3 Department of plastic and burns surgery, National Key Clinical Construction Specialty, The Affiliated Hospital of Southwest Medical University, Luzhou, Sichuan PR China; 4 Endocrinology Department, The Second People’s Hospital of Yibin‧West China Yibin Hospital, Sichuan University, Yibin, Sichuan PR China

**Keywords:** Stress signalling, Chronic inflammation

## Abstract

Diabetic wound (DW) is characterized by elevated pro-inflammatory cytokines and cellular dysfunction consistent with elevated reactive oxygen species (ROS) levels. Recent advances in immunology have dissected molecular pathways involved in the innate immune system where cytoplasmic DNA can trigger STING-dependent inflammatory responses and play an important role in metabolic-related diseases. We investigated whether STING regulates inflammation and cellular dysfunction in DW healing. We found that STING and M1 macrophages were increased in wound tissues from DW in patients and mice and delayed the wound closure. We also noticed that the massively released ROS in the High glucose (HG) environment activated STING signaling by inducing the escape of mtDNA to the cytoplasm, inducing macrophage polarization into a pro-inflammatory phenotype, releasing pro-inflammatory cytokines, and exacerbating endothelial cell dysfunction. In Conclusion, mtDNA-cGAS-STING pathway activation under diabetic metabolic stress is an important mechanism of DW refractory healing. While using STING gene-edited macrophages for wound treatment by cell therapy can induce the polarization of wound macrophages from pro-inflammatory M1 to anti-inflammatory M2, promote angiogenesis, and collagen deposition to accelerate DW healing. STING may be a promising therapeutic target for DW.

## Introduction

Diabetes is one of the most urgent global health emergencies of the 21st century. More and more people are living with this condition, which can have life-changing complications. According to the IDF global report in 2021, DW has been one of the most severe and expensive treatment complications of diabetes and affects 40–60 million people [1]. Diabetes patients are at a 30% lifetime risk of developing an ulcer, and up to 85% of all lower-limb amputations are preceded by foot ulcers [2]. Although the therapeutic options available for DW and our knowledge of the mechanisms of DW healing have improved considerably over the past few decades, the underlying molecular mechanisms remain not fully understood. Recent advances in immunology have dissected molecular pathways involved in the innate immune system that induce innate immune cells to engage and regulate inflammatory responses [3, 4]. A growing body of evidence suggests that the interaction between immune and metabolic responses is essential for maintaining tissue and organ homeostasis [5].

In the state of DW, the abnormal epithelial barrier is caused by changes in the microbiome located in the skin and their metabolites, environmental damage, or the genetic tendency of the host, as well as the cellular contents released after necrosis and cell membrane destruction caused by stress, injury and metabolic pressure, recruit and activate innate immune cells and initiate innate immunity not only through intracellular Damage-Associated Molecular Patterns (DAMPs) but also through extracellular DAMPs and Pathogen-Associated Molecular Patterns (PAMPs) released by extracellular matrix recruitment [6, 7]. When the activated innate immunity plays a role in removing pathogens and necrotic tissues, the high concentration of Reactive Oxygen Species (ROS) produced also causes lipid peroxidation damage to the cell membrane, increases membrane permeability, destroys the critical balance of ion concentration inside and outside the cell, and further aggravate the release of DAMPs [8]. This subtle relationship as initiating factors regulate the balance between innate immunity and pro-inflammatory microenvironment and participate in the process of DW healing. As one of the most studied cell types among innate immune cells. Macrophages are of particular concern. It undergoing a phenotypic change from quiescence to activation during DW healing, playing a role in the triggering and maintenance of inflammation important role [9]. A large number of clinical studies and in vivo and in vitro studies have pointed out that the presence of excessive infiltrating macrophages and pro-inflammatory M1 phenotype macrophages in DW is the root cause of chronic wound inflammation [10]. These pro-inflammatory M1 macrophages persistently release multiple pro-inflammatory cytokines, including IL-1β, and exacerbate DW healing impairment. These findings demonstrate the critical role of the innate immune system and especially macrophages in DW healing.

cGAS-STING signaling is initially thought to activate innate immunity by identifying DNA derived from microorganisms such as viruses or bacteria. However, existing studies have shown that under certain pathological conditions, this signaling pathway can also sense cytoplasmic DNA as a cellular danger signal. Cytoplasmic DNA triggers a STING-dependent inflammatory response and is associated with a variety of severe auto-inflammatory and immune diseases in humans. In addition, mitochondrial dysfunction or abnormal activation of cGAS-STING under metabolic stress can also induce more common diseases, such as obesity, obesity-induced inflammation, insulin resistance, etc [11]. These were associated with cGAS-STING activation of excessive interferon and/or inflammatory gene expression. More and more studies have found that cGAS-STING signaling is involved in many physiological and pathological processes. DNA-activated cGAS uses ATP and Guanosine Triphosphate (GTP) as substrates to generate the second messenger cGAMP, which binds and activates the interferon gene STING. The activated STING further recruits TBK1 and initiates two downstream signaling pathways, TBK1-IRF3-IFN and TBK1-TRAF6-NF-κB, which mainly mediate inflammatory responses, can cause a wide range of inflammatory responses and defense mechanisms [12]. Recent studies have found that the leakage of autologous genomic DNA or mtDNA into the cytoplasm and abnormal activation of cGAS-STING signaling may be an important mechanism for the occurrence and development of inflammatory diseases [13]. DNA released after pancreatic acinar cell death activates STING signaling in macrophages and promotes the expression of downstream TNF-α and IFN-β, leading to increased inflammation and pancreatic damage. DMXAA-induced STING activation can further aggravate acute pancreatitis, whereas Inhibition of STING activation by DNase-I degradation of DNA released from necrotic acinar cells ameliorated the progression of acute inflammation [14]. Further studies found that STING deficiency can reduce the M1 phenotype of macrophages [15]. While palmitic acid (PA) activates the cGAS-STING-IRF3 pathway, it also inhibits angiogenesis by inducing endothelial cell inflammation [16]. These all show the therapeutic promise of targeting cGAS-STING signaling in inflammatory diseases and tissue repair. Given this, we urgently need to elucidate the exact role of STING in DW healing. In this study, our findings suggest a new mechanism involving the innate immune system STING played a detrimental role in DW healing. We propose that this effect of STING impairing DW healing may be mediated by enhancing the macrophages M1 pro-inflammatory phenotype and provide a strategy to target STING for DW disease. In this study, we hypothesized that STING signaling exacerbated wound inflammation and thereby worsened DW healing. To test our hypothesis, we first examined the expression of STING in clinical DW lesion specimens. In vivo studies were conducted in diabetic mice and diabetic mice treated with STING agonists and inhibitors to further elucidate the effect of regulating STING on DW healing. Given that the pro-inflammatory phenotype of macrophages is the basic feature of DW refractory, we selected BMDM and tested its phenotypic changes after STING activation and silencing through in vitro experiments to further verify our conjecture. We finally used STING gene-edited macrophages for wound treatment by cell therapy and observed the effect on DW healing to demonstrate the role of STING signaling on DW healing and obtain Clinical transformation evidence. Taken together, our findings suggested a new mechanism involving the innate immune system STING involved in DW healing. And obtained evidence for the detrimental role of STING in the healing process of DW. We proposed that this effect of STING impairing DW healing may be mediated by enhancing the macrophages M1 pro-inflammatory phenotype and proposed a strategy to target STING for DW disease.

## Results

### STING excessively activates in macrophages of human patients with DW

**Fig. 1:**
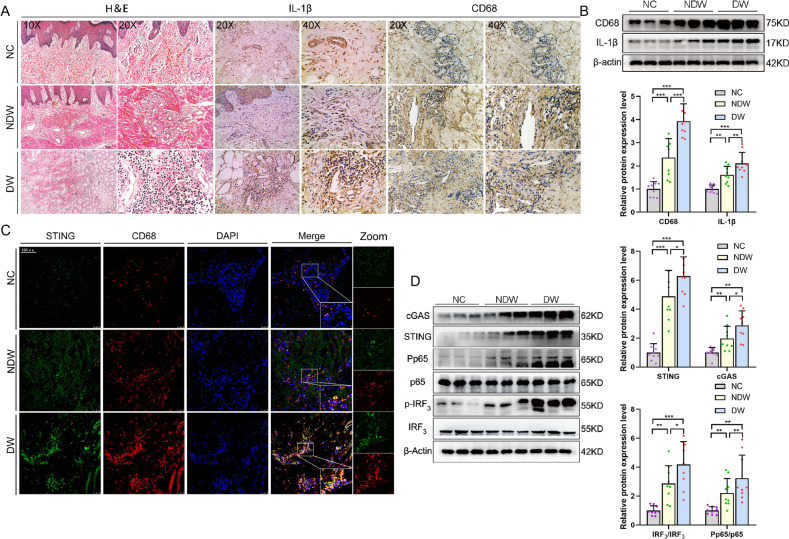
STING and its downstream effectors expression are excessively increased in macrophages of human patients with DW. **A** Sections of NC (upper row), NDW (middle row), and DW (lower row) were stained with H&E (Columns 1 and 2 on the left) or for IL-1β expression (Columns 3 and 4 on the left) and CD68 expression (right 2 columns). **B** Skin and wound lysates were examined for IL- 1β and CD68 using western blot analysis. Blots are quantified using bar graphs. **C** Sections of NC (upper row), NDW (middle row), and DW (lower row) were stained with co-localization of macrophages and STING using laser confocal microscopy. Anti-CD68 antibody labeled Macrophages (red), anti-STING antibody labeled STING (green) and DAPI labeled nucleus (blue). **D** Skin and wound lysates were examined for cGAS, STING, and inflammatory signaling using western blot analysis. Blots are quantified using bar graphs. For all bar graphs, Data were represented as mean ± SD (*n* = 9). *P < 0.05, **P < 0.01, and ***P < .001, NC vs NDW, NC vs DW, NDW vs DW.

STING has previously been shown to be a critical link between innate and adaptive immunity and activate interferon (IFN) and inflammatory cytokine responses [17]. To expound on the relevance of STING to human patients with DW, we first detected CD68 and IL-1β expression in NC, NDW, and DW groups. Compared with NC, wounded tissues showed increased staining intensity indicated by Hematoxylin-eosin Staining (H&E) and Immunohistochemistry Stain (IHC). We have also found that the recruitment of macrophages and the infiltration of inflammatory cytokine IL-1β in wounded tissues from DW was significantly stronger than that of NDW (Fig. 1A). Meanwhile, proteins in each group were determined by Western blot analysis. The results demonstrated that the expression levels of CD68 and IL-1β from wounded tissues, especially from DW expressed much stronger than that from NC (Fig. 1B). These results suggested that the diabetic environment exacerbated macrophages recruitment and inflammatory infiltration in trauma. STING is mainly expressed in the rough endoplasmic reticulum, mitochondria, and outer membrane of microsomes of human Macrophages, T lymphocytes, Dendritic cells, Endothelial cells, Epithelial cells, and Fibroblasts. To anchor key cells activated by STING in human patients with DW, we detected the co-localization of STING and macrophages in each group by Laser Confocal Microscopy. The results demonstrated that the staining intensity and co-localization of STING and macrophages (CD68^+^ positive cells) from wounded tissues, especially from DW expressed much stronger than that from NC (Fig. 1C). Meanwhile, proteins in each group were determined by Western blot analysis. The results demonstrated that the expression levels of cGAS, STING, and the phosphorylation states of IRF3 and NF-κB p65 from wounded tissues, especially from DW were significantly stronger than that from NC (Fig. 1D). These results suggested that activation of STING and its downstream effectors in macrophages aggravated the healing disorder of human patients with DW.

### Wound healing is coupled with increased signaling events of STING

**Fig. 2:**
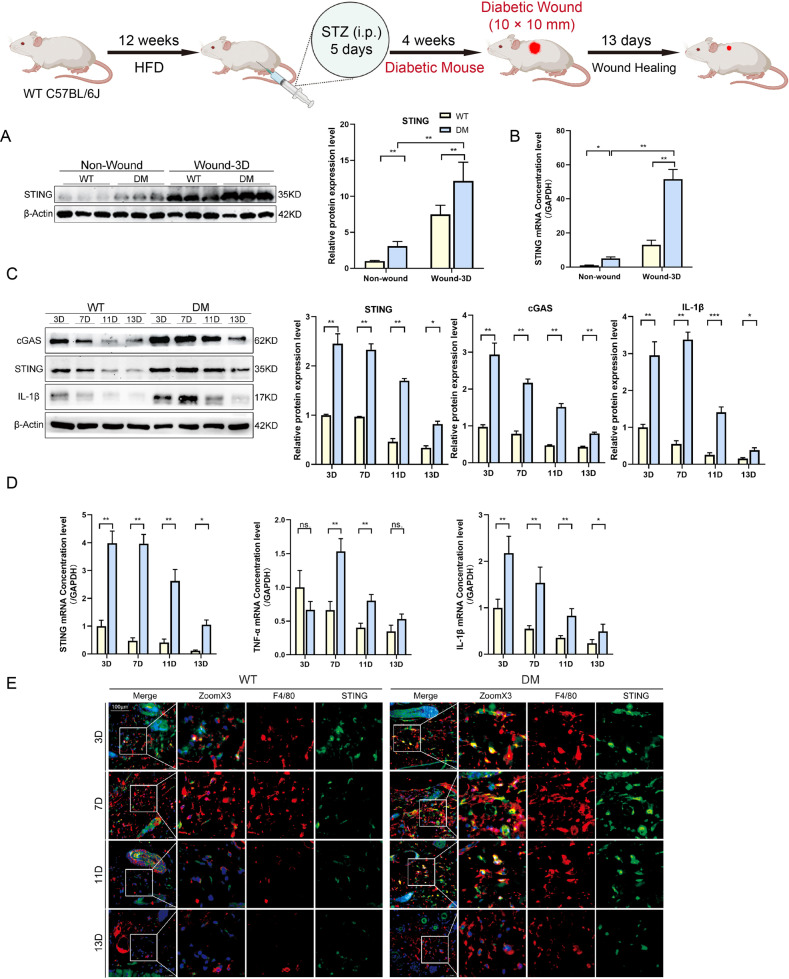
Wound healing is coupled with increased signaling events of STING. Male WT C57BL/6 J mice, at 4–5 weeks of age, were fed an HFD for 12 weeks, then intraperitoneally injected Streptozocin (STZ) to form a diabetes model (DM mice), 4 weeks later, all WT and DM mice were prepared 10 × 10 mm2 wounds on the backs using skin punches, then waited for natural healing. **A** Non-wounded and wounded back skin lysates (days 3 after trauma, indicated at the top of each lane) of WT and DM mice were examined for STING using western blot analysis. Blots are quantified using bar graphs. **B** Skin and wound mRNA levels were examined using RT-qPCR. **C** Non-wounded and wounded back skin lysates (days 3, 7, 11, 13 after trauma, indicated at the top of each lane) of WT and DM mice were examined for cGAS, STING, and IL-1β using western blot analysis. Blots are quantified using bar graphs. **D** Wound mRNA levels were examined using RT-qPCR. **E** Sections of WT (left 4 columns) and DM (right 4 columns) on days 3, 7, 11, and 13 after trauma (upper, middle 1–2, and lower row) were stained with colocalization of macrophages and STING using living cell imaging microscopy. Anti-F4/80 antibody labeled Macrophages (red), anti-STING antibody labeled STING (green) and DAPI labeled nucleus (blue). For all bar graphs, Data were represented as mean ± SD (*n* = 3). *P < 0.05, **P < 0.01 and ***P < 0.001, WT vs DM.

To investigate the role of STING in wound healing in vivo, we compared wound lesion progression in WT and DM groups (Fig. 2 Schematic diagram). We first detected the protein levels in non-wounded and wounded tissues with or without diabetes by Western blot analysis. Compared with Non-wounded, wounded tissues showed increased levels of STING, we have also found that diabetes further accentuated STING expression (Fig. 2A). Similarly, RT-qPCR presented that STING mRNA expression was also stronger in wounded tissues, especially in DW (Fig. 2B). To further investigate the effect of diabetes on wound lesion progression, we continued to detect the cGAS, STING, and IL-1β protein levels in wounded tissues at 3, 7, 11, and 13 days after trauma with or without diabetes by Western blot analysis. The results demonstrated that the expression levels of cGAS, STING, and IL-1β gradually decreased with healing in WT, but until 11 days after trauma, it still maintained a higher expression level in DM (Fig. 2C). Similarly, RT-qPCR presented that STING and proinflammatory-related mRNA (IL-1β and TNF-α) expression was also stronger in DW (Fig. 2D). In addition, we detected the co-localization of STING and macrophages in each group by Living Cell Imaging Microscopy. The results demonstrated that the staining intensity and co-localization of STING and macrophages (F4/80^+^ positive cells) from DM expressed much stronger than that from WT, especially at 7 and 11 days after trauma (Fig. 2E). These results suggested that the diabetic environment exacerbated STING sustaining activation, and the phenomenon was more pronounced after trauma.

### Treatment with STING inhibitor/agonist promotes/worsens DW healing

**Fig. 3:**
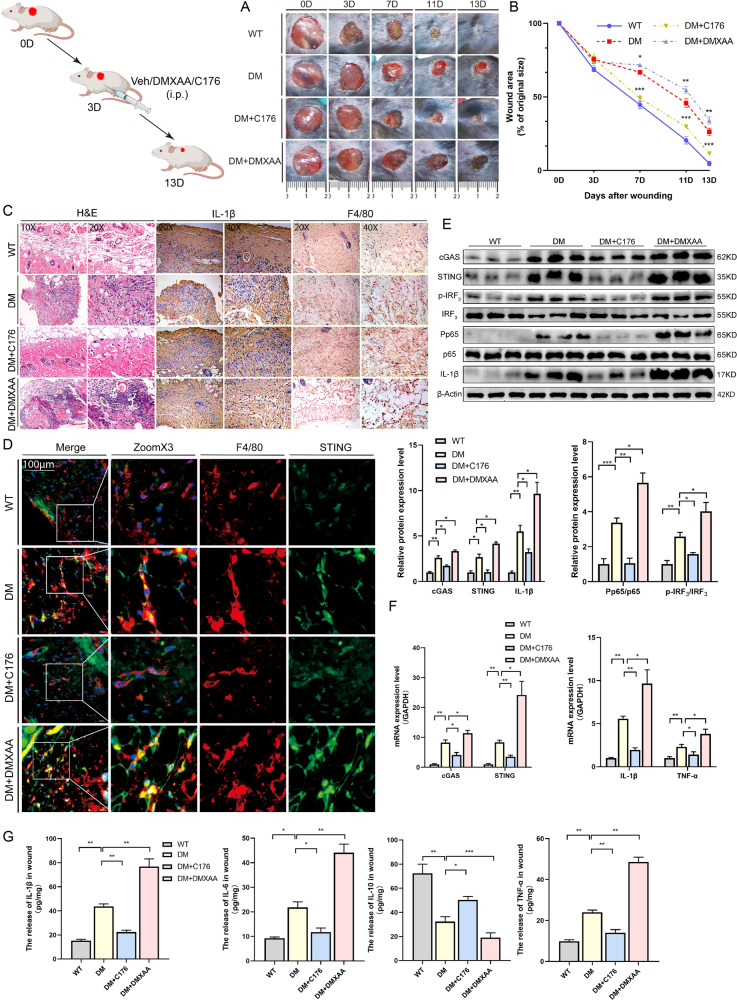
Treatment with STING inhibitor/agonist promotes/worsens DW healing. All WT and DM mice were prepared 10 × 10 mm2 wounds on the backs using skin punches, 3 days after trauma, all DW mice were intraperitoneally injected with Vehicle, DMXAA, and C-176. **A** Representative images of wounded skin after treatment with either vehicle or DMXAA and C-176 at days 0, 3, 7, 11, and 13 after trauma. **B** Percent of wound area at each time following vehicle or DMXAA and C176 treatment relative to the original wound area. Quantification of wound areas in *n* = 6 (Veh, DMXAA, and C176) wounds per group were performed with Fiji software. Sections of WT, DM, DM + C176, and DM + DMXAA (upper, middle 1–2, and lower row) at days 7 after trauma were stained with H&E (Columns 1 and 2 on the left) or for IL-1β expression (Columns 3 and 4 on the left) and F4/80 expression (right 2 columns) (**C**), and colocalization of macrophages and STING using living cell imaging microscopy. Anti-F4/80 antibody labeled Macrophages (red), anti-STING antibody labeled STING (green) and DAPI labeled nucleus (blue) (**D**). **E** Wound lysates (days 7 after trauma) of all groups (indicated at the top of each lane) were examined for cGAS, STING, IL-1β, and inflammatory signaling using western blot analysis. Blots are quantified using bar graphs. **F** Wound mRNA (days 7 after trauma) levels were examined using RT-qPCR. **G** The secretion levels of inflammatory cytokines IL-1β, TNF-α, IL-6, and IL-10 in the wound homogenates (days 7 after trauma) were detected by ELISA. For all bar graphs, Data were represented as mean ± SD (*n* = 6). *P < 0.05, **P < 0.01 and ***P < 0.001, DM vs DM + DMXAA, DM vs DM + C176 (in **B**), WT vs DM, DM vs DM + DMXAA, DM vs DM + C176 (in **E**–**G**).

As described above, STING and its downstream effectors were excessively activated in DW, which suggested that STING signaling played an important role in DW healing. As a further confirmation, we used a pharmacologic agent to induce STING deficiency or activation. Previous reports show that C-176 is a STING inhibitor and DMXAA is a STING agonist. We tested whether STING is up-regulated and its absence leads to protection from DW healing (Fig. 3 Schematic diagram). We first observed the wound closure by measuring the change in the wound area and checking the macroscopic differences in the wounds (Fig. 3A). The closure rate of the wound was not significantly different 3 days after trauma. Then we evaluated the effect of the STING inhibitor (DM + C176) group or the agonist (DM + DMXAA) group on the wounds of the DM models, using the Vehicle as control (DM) group. As expected, compared with DM, DM + C176 exhibited significantly higher percentages of wound closure at 7, 11, and 13 days after trauma, while DM + DMXAA showed a worsened healing trend (Fig. 3B). In addition, compared with DM, DM + C176 showed decreased staining intensity indicated by H&E and IHC, while DM + DMXAA showed significantly increased staining intensity (Fig. 3C). We also detected the co-localization of STING and macrophages in each group by Living Cell Imaging Microscopy. The results demonstrated that the staining intensity and co-localization of STING and macrophages from DM + C176 expressed weaker than that from DM, which was reversed by DM + DMXAA (Fig. 3D). These results showed that STING agonist also elevated the number of macrophages in wound tissues from DM, while the addition of C-176 inhibited this effect. Lastly, we detected the protein levels in each group by Western blot analysis. The results demonstrated that the expression levels of cGAS, STING, IL-1β, and the phosphorylation states of IRF3 and NF-κB p65 from DM + C176 were significantly decreased than that from DM, while the opposite trend was observed in DM + DMXAA (Fig. 3E). Similarly, RT-qPCR presented that cGAS, STING, and Inflammation-related mRNA (IL-1β and TNF-α) expression was also stronger in DM + DMXAA while the addition of C-176 inhibited this effect (Fig. 3F). When wound inflammatory status was examined by ELISA, the proinflammatory cytokine IL-1β, IL-6, TNF-α were decreased, and the anti-inflammatory cytokine IL-10 was increased in DM + C176. Consistent with the results in which inhibition of STING with C-176 led to amelioration of DW healing, activation of STING with DMXAA led to worsening of DW healing (Fig. 3G). These results suggested that STING played an important role in mediating inflammation during DW healing and overexpression of STING in vivo in macrophages exacerbated the wound healing disorder in diabetics.

### High glucose (HG) via ROS induces mitochondrial DNA (mtDNA) leakage and activates STING signaling in macrophages

**Fig. 4:**
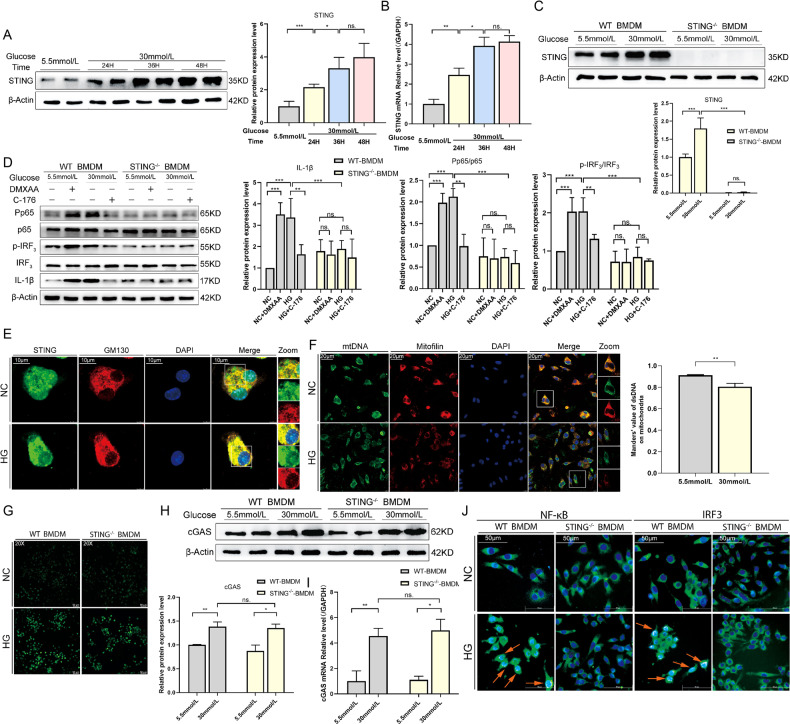
HG via ROS induces mitochondrial DNA (mtDNA) leakage and activates STING signaling in macrophages. **A** BMDM were treated with HG (30 mmol/L) for 24, 36, and 48 h or normal control (NC, 5.5 mmol/L) for 24 h, then examined for STING using western blot analysis. **B** The mRNA levels were examined using RT-qPCR. **C** BMDM from STING^−/−^ and WT C57BL/6 J mice (WT-BMDM and STING^−/−^-BMDM) were treated with HG and NC for 24 h, then examined for STING using western blot analysis. **D** WT-BMDM and STING^−/−^-BMDM were treated with or without DMXAA (75 mg/mL) and C176 (5 nmol/mL) for 24 h in the absence or presence of HG for the last 24 h, then examined for inflammatory signaling using western blot analysis. BMDM were treated with HG and NC for 24 h, then examined for the displacement of STING toward Golgi apparatus (GM130) and mtDNA leakage using laser confocal microscopy. Anti-GM130 antibody labeled Golgi apparatus (red), anti-STING antibody labeled STING (green) and DAPI labeled nucleus (blue) (**E**), **F** Anti-Mitofilin antibody labeled Mitochondria (red), anti-dsDNA antibody labeled mtDNA (green) and DAPI labeled nucleus (blue). WT-BMDM and STING^−/−^-BMDM were treated with HG and NC for 24 h, then examined for ROS production using living cell imaging microscopy (**G**), cGAS protein expression using western blot analysis (**H**), mRNA levels using RT-qPCR (**I**), downstream NF-κB and IRF3 activation using living cell imaging microscopy (**J**). For all bar graphs, Measurement Data were represented as mean ± SD. One-way analysis of variance (ANOVA) and Tukey’s post hoc test were used for comparing data among multiple groups. The cell experiment was repeated 3 times. *P < 0.05, **P < 0.01 and ***P < 0.001, NC vs HG (in **A**, **B**, **C**, **H**, and **I**), NC vs NC + DMXAA, HG vs HG + C176 and WT-BMDM vs STING^−/−^-BMDM in HG condition (in **D**).

Based on our finding that excessive activation of STING was critical for promoting inflammation in DW healing and macrophages were the most prominent up-regulators of STING. We treated bone marrow-derived macrophages (BMDM) with HG to recapitulate our in vivo findings and gain mechanistic insights. In WT BMDM, 30 mM of glucose treatment caused a significant increase in STING expression in a time-dependent manner detected by Western blot analysis (Fig. 4A) and RT-qPCR (Fig. 4B), confirming our previous finding. Strikingly, in STING^−/−^ BMDM, STING protein levels were not altered under basal or 30 mM glucose-stimulated conditions (Fig. 4C). To further study the effect of HG-induced STING activation in pro-inflammatory responses of macrophages. We then treated WT and STING^−/−^BMDM with DMXAA to activate STING. Interestingly, WT BMDM, but not STING^−/−^ BMDM, produced significantly more IL-1β and increased phosphorylation states of IRF3 and NF-κB p65 as compared to HG-induced macrophages, indicating that STING activation promotes pro-inflammatory responses of WT macrophages. In addition, We treated WT and STING^−/−^BMDM with C-176, we also found that STING deficiency, as well as knockout, reduced the pro-inflammatory responses of macrophages induced by HG (Fig. 4D). These data were consistent with previous research, which indicated HG triggers pro-inflammatory effects in macrophages via STING.

As a DNA sensor, STING could be activated by free DNA in the cytoplasm in previous reports. We hypothesized that HG could activate ROS and cause mtDNA leakage into the cytoplasm. To further validate this possibility, we first observed the co-localization of STING and Golgi apparatus in WT BMDM. Based on Laser Confocal Microscopy results, STING was found to weakly co-localize with Golgi matrix protein 130 (GM130) in untreated WT BMDM. Conversely, HG promoted the displacement of STING toward the Golgi apparatus (GM130), indicating activation of STING (Fig. 4E). In addition, the co-localization of mtDNA (dsDNA) and Mitochondria (Mitofilin) showed that dsDNA was located in mitochondria in the untreated WT BMDM, while HG damaged the morphology of mitochondria and escaped dsDNA into the cytoplasm (Fig. 4F). Furthermore, the generation of ROS was triggered by HG regardless of STING expression as predicted (Fig. 4G). These results suggested that HG-induced cytoplasmic free dsDNA originates at least in part from damaged mitochondria, which is inseparable from the enhanced oxidative stress in the HG environment. We further detected that HG caused a significant increase in cGAS expression in both WT and STING^−/−^ BMDM by Western blot analysis (Fig. 4H) and RT-qPCR (Fig. 4I). This further highlighted the key regulatory role of STING in the inflammatory response of macrophages. Meanwhile, immunofluorescence analyses disclosed that HG promoted the nuclear translocation of IRF3 and NF-κB p65 in WT BMDM which disappeared in STING^−/−^ BMDM (Fig. 4J). Taken together, these findings revealed that HG activated STING signaling in BMDM by releasing ROS to induce mtDNA leakage, and STING played a key regulatory role in HG-induced macrophages inflammatory response.

### STING activation promotes M1 polarization of macrophages in DW healing

**Fig. 5:**
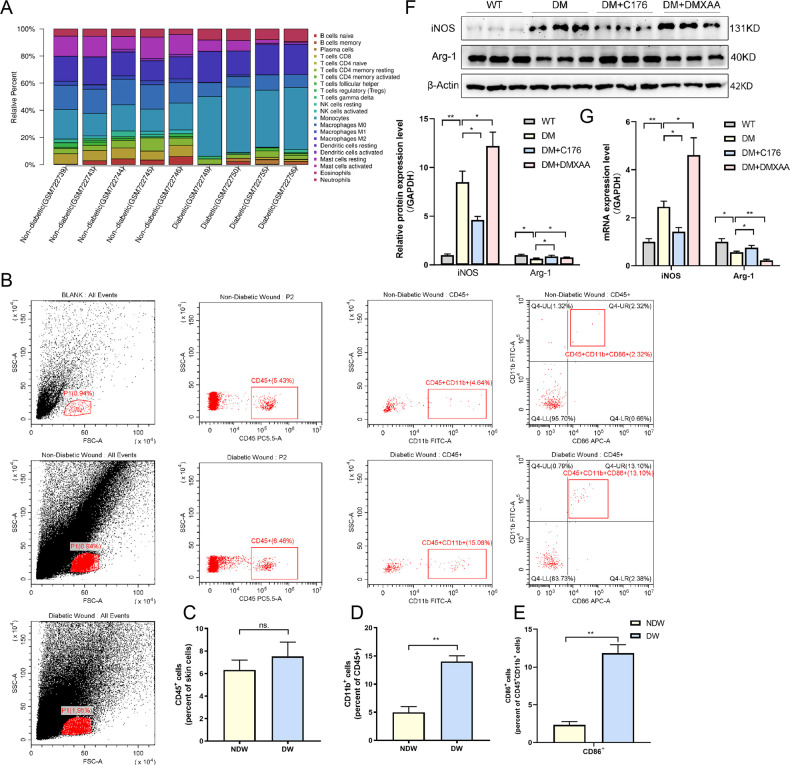
STING activation promotes M1 polarization of macrophages in DW healing. **A** Differential immune cell infiltration patterns between normal and diabetic tissue microenvironments were performed using GEO microarrays. **B** Wound leukocytes were isolated for flow cytometry and scatter plots, as well as those gated on wound total leukocytes (CD45), are shown. **C** Percentage of CD45^+^ cells in total wound homogenate in NDW and DW. **D** Percentage of CD11b^+^ cells in total wound leukocytes (CD45 ^+^ cells) in NDW and DW. **E** Percentage of CD86^+^ cells in total wound macrophages (CD45 ^+^ CD11b^+^ cells) in NDW and DW. Data were represented as mean ± SD from at least 3 independent experiments (*n* = 3 patients per group and experiment). **F** Wound lysates (at days 7 after trauma) of all groups (indicated at the top of each lane) were examined for iNOS and Arg-1 using western blot analysis. Blots are quantified using bar graphs. **G** Wound mRNA (at days 7 after trauma) levels were examined using RT-qPCR. For all bar graphs, data were represented as mean ± SD (*n* = 5). *P < 0.05, **P < 0.01 and ***P < 0.001, NDW vs DW (in **C**–**E**), WT vs DM, DM vs DM + DMXAA, DM vs DM + C176 (in **F**–**G**).

Accumulating evidence has shown that macrophages polarization plays a critical role in DW healing. Therefore, based on the Gene Expression Matrix of Normal and Diabetic Tissues (Source from GENE EXPRESSION OMNIBUS, GPL6947 Illumina HumanHT-12 V3.0 expression bead chip), we used the online web tool CIBERSORT (http://cibersort.stanford.edu/) to characterize the expression of immune cell phenotypes of 22nd different cell types and functional states from the perspective of immune infiltration to obtain differential expression data, Our prediction results found that there were differences in the infiltration of macrophages, monocytes, neutrophils, natural killer cells, and mast cells (Fig. 5A). Furthermore, We analyzed leukocytes cells expression (CD45^+^ positive) in human patients with DW and Non-DW. Although there was no significant difference in leukocyte expression in DW and NDW tissues (Fig. 5B, C), an increase in macrophages-positive (CD11b^+^ positive) was observed in DW (Fig. 5B, D) with M1 polarization (CD86^+^ positive) of accounting for most of the expression of the macrophages (Fig. 5B, E). These results suggested that macrophages polarized to M1 in DW healing. Based on the previous conclusion, we speculated that STING activation could affect macrophages polarization in wound healing. To further examine STING signaling in macrophages polarization, we sorted macrophages from WT, DM, DM + C176, and DM + DMXAA mice wound tissue. Macrophage M1/M2 polarization status was determined by RT-qPCR and Western blot analysis. The results demonstrated that macrophages from DM expressed a significantly higher level of M1 macrophage marker (iNOS) and lower level of M2 macrophage marker (Arg-1) compared with that from WT, while DMXAA activated STING enhanced the absolute M1 macrophages numbers, which was reversed by C-176 (Fig. 5F, G). Taken together, these findings revealed that activation STING signaling elevated the number of M1 macrophages in wound healing.

### Activated STING enhances pro-inflammatory responses and vascular endothelial dysfunction

**Fig. 6:**
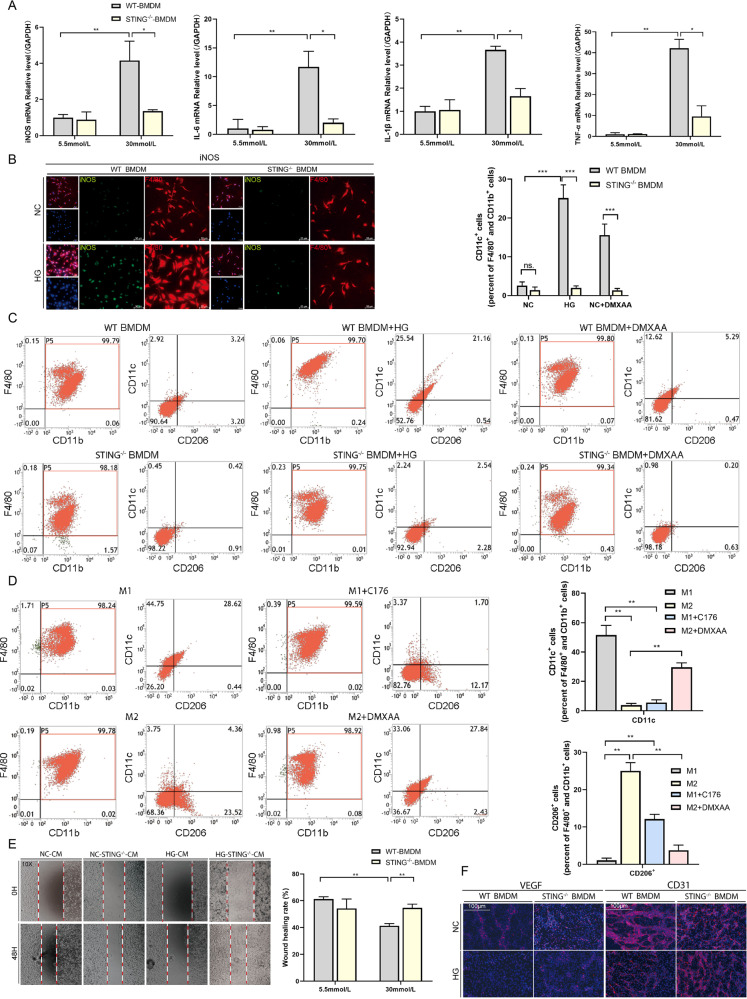
STING enables macrophages to M1 polarization that promotes pro-inflammatory responses and vascular endothelial cell dysfunction. **A** WT-BMDM and STING^−/−^-BMDM were treated with HG and NC for 24 h, then the mRNA levels were examined using RT-qPCR (**B**) WT-BMDM and STING^−/−^-BMDM were treated with HG and NC for 24 h, then examined for colocalization of iNOS and F4/80 using living cell imaging microscopy. Anti-F4/80 antibody labeled Macrophages (red), anti-iNOS antibody labeled M1macrophages (green) and DAPI labeled nucleus (blue). **C** WT-BMDM and STING^−/−^-BMDM were treated with or without DMXAA for 24 h in the absence or presence of HG for the last 24 h for flow cytometry and scatter plots, as well as those gated on M1 macrophages (F4/80 + CD11b + CD11c + CD206- cells), were shown. **D** WT-BMDM were treated with LPS (50 ng/ml) +IFN-γ (50 ng/ml) (M1), IL-4 (40 ng/ml) (M2) for 24 h, then treated with C-176 (M1 + C176) or DMXAA (M2 + DMXAA) respectively for 24 h for flow cytometry and scatter plots, as well as those gated on M1 macrophages (F4/80^+^CD11b^+^CD11c^+^CD206^−^ cells) and M2 macrophages (F4/80^+^ CD11b^+^CD206^+^ CD11c^−^ cells) were shown. **E** WT-BMDM and STING^−/−^-BMDM were treated with HG and NC for 24 h, collected cell supernatant, mixed with the fresh medium at a ratio of 1:1, and added to RVEs, then observed the migration ability by scratch wound assay. **F** VEGF and CD31 were Observed using living cell imaging microscopy. For all bar graphs, Data were represented as mean ± SD. The cell experiment was repeated 3 times. *P < 0.05, **P < 0.01 and ***P < 0.001, WT-BMDM vs STING^−/−^-BMDM (in **A**, **C**, **E**, and **F**), M1 vs M2, M1 vs M1 + C176, M2 vs M2 + DMXAA (in **D**).

Our present results demonstrated that DMXAA treatment increased the number of M1 macrophages in wounded tissue in vivo, supporting the theory that STING activation-induced macrophages toward M1 polarization. To confirm this finding in vitro, BMDM isolated from the bone marrow of mice was used. In WT BMDM, HG treatment caused a significant increase in iNOS and pro-inflammatory mRNA (IL-6, IL-1β, and TNF-α) expression detected by RT-qPCR, confirming our previous finding. However, in STING^−/−^BMDM, HG treatment did not lead to similar changes (Fig. 6A). Meanwhile, immunofluorescence analyses disclosed that HG promoted M1 polarization (iNOS) in WT BMDM which reversed in STING^−/−^ BMDM (Fig. 6B). We used F4/80^+^CD11b^+^CD11c^+^CD206^−^ to define M1 macrophages and F4/80^+^CD11b^+^CD206^+^CD11c^−^ to define M2 macrophages, the results showed that DMXAA treatment induced M1 polarization in macrophages consistent with HG stimulation, and STING silencing exhibited the ability to reverse HG-induced M1 polarization in macrophages (Fig. 6C). We further induced BMDM polarization to M1 or M2 with LPS (50 ng/ml) + IFN-γ (50 ng/ml) or IL-4 (40 ng/ml), then treated with C-176 and DMXAA respectively. The results showed that DMXAA activated STING to promote M2 macrophages polarization to M1, while C-176 inhibited STING to induce M1 macrophages polarization to M2 (Fig. 6D).

Insufficient angiogenesis is an important reason for refractory DW. We further explored whether STING regulation affects angiogenesis through macrophages. We cultured WT and STING^−/−^ BMDM under NC and HG conditions. Collected the supernatant and mixed the fresh medium at a ratio of 1:1 and added them into MVEs mixed with the fresh medium at a ratio of 1:1 to observe the effect on the function of vascular endothelial cells. The results showed that HG-CM downregulated the migratory capacity of vascular endothelial cells, while HG-STING^−/−^-CM reversaled this phenomenon (Fig. 6E). VEGF and CD31 are important growth factors affecting angiogenesis. We then observed the effects of WT and STING^−/−^ BMDM supernatants under NC and HG conditions on VEGF and CD31 secretion in MEVs by Living cell imaging microscopy. The results showed that HG-CM down-regulated the expressions of VEGF and CD31 in MVEs, while HG-STING^−/−^-CM up-regulated the expressions of VEGF and CD31 (Fig. 6F). Taken together, these findings suggested that STING deficiency could ameliorate the effect of HG-induced macrophages on vascular endothelial cell dysfunction. This might be related to the induction of macrophages polarization from the pro-inflammatory M1 phenotype to the M2 phenotype.

### Cell therapy regulates macrophages STING disruption accelerates DW healing

**Fig. 7:**
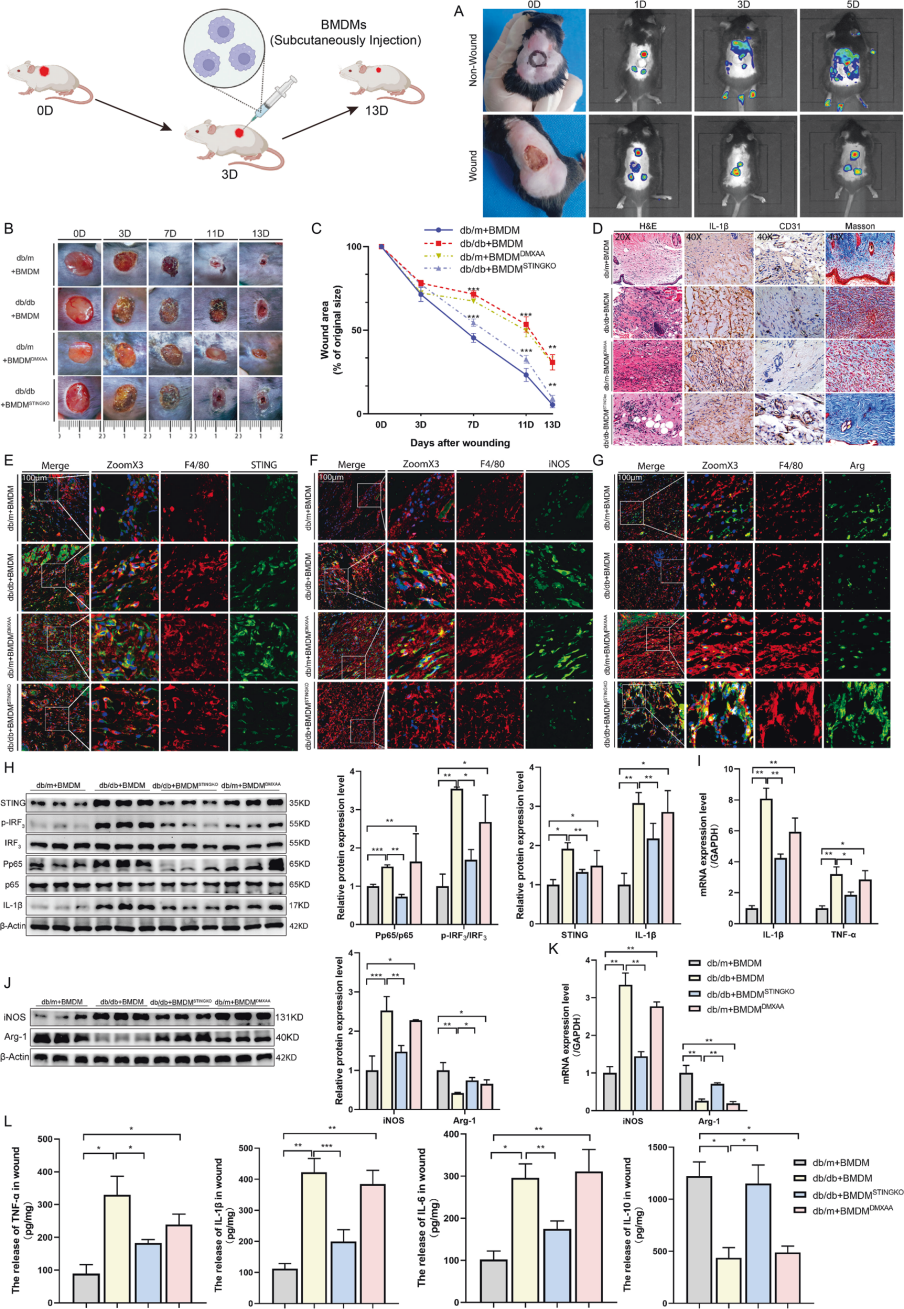
Cell therapy regulates macrophages STING disruption accelerates DW healing. All db/m and db/db mice were prepared 10 × 10 mm2 wounds on the backs using skin punches, after an injury on the 3rd day, all mice were subcutaneously injected BMDMs around the wound edge. **A** Colonization of BMDMs after subcutaneous injection at days 0, 1, 3, and 5 after wounding using a Small animal imaging system in vivo. **B** Representative images of wounded skin after treatment with BMDMs at days 0, 3, 7, 11, and 13 after wounding. **C** Percent of wound area at each time following BMDMs treatment relative to the original wound area. Quantification of wound areas were performed with Fiji software. Sections of db/m+BMDM, db/db+BMDM, db/m+BMDM^DMXAA^, and db/db+BMDM^STINGKO^ (upper, middle 1–2, and lower row) at days 7 after trauma were stained with H&E (Columns 1 on the left) or for IL-1β and CD31 expression (Columns 2 and 3 on the left) and Masson expression (right 1 column). **D** Colocalization of macrophages and STING, iNOS and STING, Arg-1 and STING using living cell imaging microscopy. Anti-F4/80 antibody labeled Macrophages (red), anti-STING antibody labeled STING (green), anti-iNOS antibody labeled M1 (green), anti-Arg-1 antibody labeled M2 (green) and DAPI labeled nucleus (blue) (**E**–**G**). Wound lysates (day 7 after injury) of all groups were examined for STING, IL-1β (**H**), iNOS, and Arg-1 (**J**) using western blot analysis (**H**) and RT-qPCR (**I**–**K**), secretion levels of inflammatory cytokines IL-1β, TNF-α, IL-6, and IL-10 by ELISA (**L**). For all bar graphs, Data were represented as mean ± SD (*n* = 5). *P < 0.05, **P < 0.01 and ***P < 0.001, db/db+BMDM vs db/db+BMDM^STINGKO^, db/m+BMDM vs db/m+BMDM^DMXAA^ (in **C**), db/m+BMDM vs db/db+BMDM, db/db+BMDM vs db/db+BMDM^STINGKO^, db/m+BMDM vs db/m+BMDM^DMXAA^ (in **H**–**L**).

As described above, STING could regulate the polarization state of macrophages. We used cell therapy technology to return STING-knockout macrophages (BMDM^STINGKO^) to the wound and studied its effect on DW healing (Fig. 7 Schematic diagram). We first found that local injection of BMDM in Non-wounded mice produced a homing phenomenon, while in wounded mice, local injection of BMDM accumulated in the center of the wound on the 3rd day after injection, and still be detected on the wound surface until the 5th day (Fig. 7A). It suggested that BMDM injected subcutaneously through the wound edge could effectively reach the treatment site. We then observed the wound closure by measuring the change in the wound area and checking the macroscopic differences in the wounds (Fig. 7B). The results showed that there was no significant difference in the wound healing rate on 3rd day after trauma. Compared with the db/db+BMDM group, the db/db+ BMDM^STINGKO^ group significantly improved the healing rate at 7, 11, and 13 days after the trauma. Compared with the db/m+BMDM group the db/m+BMDM^DMXAA^ group delayed the normal healing process (Fig. 7C). In addition, compared with the db/m+BMDM group, the db/db+BMDM group showed increased staining intensity indicated by H&E and IHC, while BMDM^STINGKO^ injection treatment significantly reduced the infiltration of inflammatory cells and the expression of pro-inflammatory factor IL-1β in the wound of db/db mice. In contrast, The BMDM^DMXAA^ injection treatment significantly up-regulated the infiltration of inflammatory cells and the expression of pro-inflammatory factor IL-1β in the wound of db/m mice. Angiogenesis and granulation formation are important indicators of effective wound healing. We also found that compared with the db/m+BMDM group, the blood vessels in the db/db+BMDM group were expressed in a monolayer structure, some lumen was narrowed, the wound tissue structure was disordered, and the collagen deposition was reduced. BMDM^STINGKO^ treatment significantly increased the number of new blood vessels and most of them had a double-layer structure in the wound of db/db mice. It also promoted the collagen fibers on the wound surface increased and arranged neatly in the wound of db/db mice. In contrast, after BMDM^DMXAA^ treatment, the new blood vessels lost their double-layered structure, decreased in number and lumens, and the collagen deposition also decreased in the wound of db/m mice (Fig. 7D). We further detected the co-localization of STING and macrophages in each group by living Cell Imaging Microscopy. The results showed that there was a significant difference in the expression of STING between the db/m group and the db/db group after BMDMs treatment (Fig. 7E). In addition, we detected the co-localization of iNOS and F4/80, Arg-1 and F4/80. The results showed that compared with the db/m+BMDM group, there was more macrophages infiltration in the db/db+BMDM group wounds, and the infiltrated macrophages expressed more M1 and less M2 phenotypes. BMDM^STINGKO^ injection caused these infiltrating macrophages mainly expressed M2 and rarely expressed M1 phenotypes. Similarly, BMDM^DMXAA^ injection caused these infiltrating macrophages mainly expressed M1 and rarely expressed M2 phenotypes (Fig. 7F, G). Taken together, this suggested that the STING gene-edited macrophages injection could differentially modulate the phenotype of wound infiltrated macrophages (exogenous and endogenous). Lastly, we detected the protein levels in each group by Western blot analysis. The results showed that compared with the db/m+BMDM group, STING signaling was activated in the db/db+BMDM group. BMDM^STINGKO^ injection treatment inhibited the activation of STING signaling and down-regulated the expression of pro-inflammatory factors IL-1β, and the phosphorylation status of IRF3 and NF-κB p65 in the db/db group. In contrast, BMDM^DMXAA^ injection treatment activated STING signaling and up-regulated the expression of the pro-inflammatory factor IL-1β, and the phosphorylation status of IRF3 and NF-κB p65 in the db/m group (Fig. 7H). The inflammation-related mRNA (IL-1β and TNF-α) expression detected by RT-qPCR showed a similar tendency (Fig. 3I). We also found that compared with the db/m+BMDM group, the expression of iNOS increased and the expression of Arg-1 decreased in the db/db+BMDM group. BMDM^STINGKO^ injection down-regulated the high expression of iNOS and up-regulated the low expression of Arg-1 in the db/db group. BMDM^DMXAA^ injection up-regulated the low expression of iNOS and down-regulated the high expression of Arg-1 in the db/m group by Western blot analysis (Fig. 3J) and RT-qPCR (Fig. 3K). When wound inflammatory status was examined by ELISA, the results showed that compared with the db/m+BMDM group, the secretion of M1-related pro-inflammatory cytokines IL-6, IL-1β, and TNF-α in the db/db+BMDM group increased, and the secretion of M2-related anti-inflammatory cytokine IL-10 decreased. BMDM^STINGKO^ injection could down-regulate the secretion of M1-related pro-inflammatory cytokines IL-6, IL-1β, and TNF-α, and up-regulate the secretion of M2-related anti-inflammatory cytokine IL-10 in the wounds of db/db mice. In contrast, BMDM^DMXAA^ injection could up-regulate the secretion of M1-related pro-inflammatory cytokines IL-6, IL-1β, and TNF-α, and down-regulate the secretion of M2-related anti-inflammatory cytokine IL-10 in the wounds of db/dm mice (Fig. 7L). Taken together, these findings suggested that exogenous supplementation of BMDM^STINGKO^ could accelerate DW healing by inducing M2 polarization in macrophages, promoting angiogenesis and matrix deposition. It also suggested that the activation of STING signaling in DW could promote the infiltration of the pro-inflammatory M1 phenotype, aggravated tissue damage, and thus affected wound healing.

## Discussion

In DW, the normal healing phase is stalled in the inflammatory phase, which is characterized by elevated pro-inflammatory cytokines, protease, ROS levels, and cellular dysfunction [18]. Due to impaired immune function and defective phagocytic and chemotactic activities of macrophages and granulocytes, DW is more likely to lead to excessive recruitment of inflammatory cells, production of various ROS, damage to the structure of the ECM [19], resulting in an inflammatory cascade that further impairs wound healing [20]. Therefore, understanding the molecular mechanism of chronic inflammation in DW disease is of great significance for the prevention and treatment of DW. The elucidation of the molecular mechanism of cGAS-STING signaling is major progress in the field of innate immunity in recent years [21]. In this study, we validated the relationship of STING to wound inflammation and healing in human subjects and animal models. It was concluded that the excessive activation of the STING signaling led to the increase of pro-inflammatory M1 macrophages which affected wound healing via aggravating tissue inflammation and damage. It was worth noting that we also found that STING is activated in the normal skin tissue of diabetic animals, which may also be an important cause of tissue aseptic inflammation and systemic chronic low-grade inflammation under metabolic pressure of diabetes. At the mechanistic level, our study provided new insights into the general phenomenon that HG injury activates the pro-inflammatory phenotype of macrophages from the perspective of innate immunity. Thus, our study provided convincing evidence that HG enhances the pro-inflammatory phenotype of macrophages by activating STING, which is involved in chronic inflammation and protracted healing in DW.

Wound healing is a dynamic process that requires delicate inflammatory initiation and resolution to achieve a balance between killing necrotic tissue and damaged cells and promoting tissue regeneration. During the complete cycle of wound healing, various cells involved in wound healing, as well as a variety of cytokines and growth factors are affected by the healing process and show different temporal and spatial expression differences [22]. We need a more detailed understanding of the dynamic changes of STING and its signaling during wound healing to determine its relevance to wound healing. Therefore, we examined the changing trends of STING and its downstream pro-inflammatory factors at different observation points before and after trauma in WT and DM mice. Through the observation of wound healing in mice, we found that trauma could activate STING signaling in the early stage of healing in both WT and DM mice, which might be related to the activation of innate immunity to respond to tissue damage by traumatic stress. With the wound healing, both macrophages infiltration and pro-inflammatory factor expression in WT mice showed a downward trend. We also observed that STING signaling and the phosphorylation status of IRF3 and NF-κB in wound tissue showed a downward trend consistent with wound healing. However, in the process of wound healing in DM mice, we observed that infiltrated macrophages and pro-inflammatory factors were consistently highly expressed. We also observed that STING signaling and the phosphorylation status of IRF3 and NF-κB showed an increasing trend consistent with the increased pro-inflammatory state of the wound, increased macrophages infiltration, and refractory wound healing. Taken together, these results confirm the tight association between STING signaling and DW healing.

Likewise, to illustrate the relevance of macrophages STING activation to human DW healing, we first examined the expression of STING and its downstream pro-inflammatory signals in normal, Non-DW, and DW skin. We found that trauma-induced the activation of STING and its downstream pro-inflammatory signaling in tissues. This phenomenon was more obvious in the underlying diseases of diabetes. Given the important role of macrophages in wound healing, we also further anchored the correlation of STING activation in macrophages with wound healing by colocalization. We conclude that STING and its downstream pro-inflammatory signaling were significantly activated in skin wounds with diabetes. We hypothesized that STING plays a villain role in DW healing. In other words, We hypothesized that inhibition or silencing of hyperactivated STING could restore the healing process of DW. Therefore, we treated diabetic mice with the STING agonist DMXAA and the inhibitor C-176 to further define the critical role of STING in DW healing. We provided at least three lines of evidence to support the deleterious role of activated STING in DW pathogenesis. Firstly: In a loss-of-function study, intraperitoneal injection of C-176 effectively inhibited STING expression in the wound tissue of diabetic mice compared with DM mice, and accelerated DW healing by promoting M2 polarization of macrophages. In contrast, in gain-of-function studies, intraperitoneal injection of DMXAA further activated STING expression in wound tissue compared with DM mice, worsened DW healing by aggravating macrophages infiltration and M1 polarization. These complementary results further suggested that STING played a central role in driving macrophages M1 polarization and pro-inflammatory factor production in DW healing. Secondly, we performed co-localization assays for activated STING and infiltrating macrophages in the wound, and we found that the expression of activated STING and infiltrating macrophages in the wounds of DM mice was not only significantly higher than that of WT mice, and there was enhanced co-localization between the two. While intraperitoneal injection of C-176 down-regulated the expression of STING in wound tissue, it also attenuated the co-localization of STING and macrophages. In contrast, intraperitoneal injection of DMXAA not only up-regulated the expression of STING and the infiltration of macrophages in the wound tissue showed stronger colocalization between them. This suggested that the specific contribution of macrophages to STING activation was much greater than that of other cells during DW healing. Lastly, the most crucial point was that when we finally used STING gene-edited BMDM to subcutaneously injection in the wound, although BMDM could be effectively delivered to the local wound tissue, the expression of STING expression and its downstream pro-inflammatory signaling in wound tissue showed a trend consistent with the polarization function of macrophages. Specifically, using BMDM^STINGKO^ treated db/db mice, not only the expression of STING and its downstream pro-inflammatory signals were attenuated in wound tissue, but also the co-localization of STING with macrophages was also weakly seen. Similarly, with BMDM^DMXAA^ treated db/m mice, not only the expression of STING and its downstream pro-inflammatory signals were increased in wound tissue, but also the co-localization of STING with macrophages also showed a consistent association with M1 macrophages expression. This further illustrated the unique contribution of macrophages in DW, especially M1 macrophages, to STING activation in wounds.

To recapitulate the results of our in vivo studies and gain mechanistic-level insights. We further validated the activation of the cGAS-STING signaling by HG injury, and the induction of M1 polarization in macrophages by STING activation in vitro. We extracted WT and STING^−/−^ BMDM and intervened with HG respectively. We found that HG activated STING expression and its downstream pro-inflammatory signaling in WT BMDM and induced macrophages M1 polarization. However, in STING^−/−^ BMDM, HG did not exhibit the expected pro-inflammatory and M1-polarizing effects. Likewise, when we stimulated WT BMDM with DMXAA, we observed pro-inflammatory and M1-polarizing effects consistent with HG injury, whereas treatment with C-176 effectively reversed the pro-inflammatory and M1-polarizing effects of HG injury. More importantly, when we co-cultured with vascular endothelial cells, STING-silenced BMDM not only increased the migratory capacity of vascular endothelial cells in an HG environment but also increased the secretion of growth factors associated with angiogenesis. It effectively reversed the damage to vascular endothelial cell function by pro-inflammatory macrophages. Therefore, we further pointed out the protective effect of silencing STING on the function of vascular endothelial cells damaged by HG. This also provided a theoretical basis for our later gene-edited cell therapy. Besides cGAS, When STING passed from the ER to the ERGIC, then to the Golgi and the post-Golgi vesicles, and at last degraded by the lysosome. The trafficking and degradation of STING were regulated through a variety of mechanisms [23]. Therefore, we examined the upstream and downstream mechanisms of HG-induced STING activation and found that HG-induced the release of ROS independent of STING expression, and these released ROS contributed to the damaged mitochondrial function. We also found the activation of the cGAS-STING pathway was not only manifested in the increased expression of cGAS and STING genes but also in the Golgi translocation of STING and nuclear translocation of downstream IRF3 and NF-κB p65. This is also consistent with many studies [24, 25]. Therefore, we proposed that macrophages exposed to HG had increased ROS release, mitochondrial damage, and the escape of mtDNA to the cytoplasm to activate the cGAS-STING signaling pathway and caused an inflammatory response. This role was at least partially involved in the progression of DW angiogenesis disorders and refractory disease.

In the last part of the study, we edited the expression of STING in macrophages and injected them into the wound to further observe the profound effect of regulating STING on DW healing. It was also an in vivo validation of the feasibility of clinical translation. Our final findings found that treatment of db/db mice with BMDM^STINGKO^ could inhibit wound STING and its downstream pro-inflammatory signaling, induced pro-inflammatory M1 macrophages to polarize towards anti-inflammatory M2 in the wound, and promoted angiogenesis and collagen production, deposition, which in turn accelerated DW healing.

**Fig. 8:**
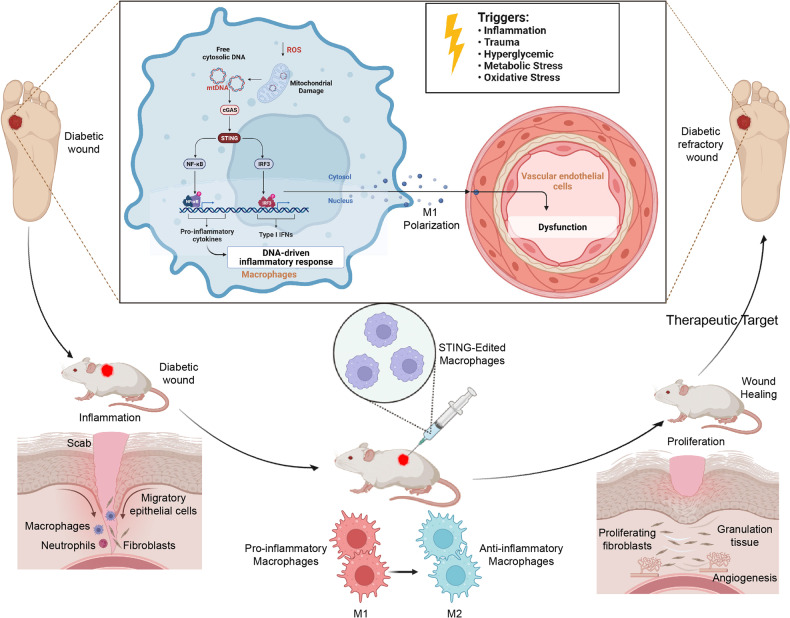
Schematic diagram showing the proposed mechanism that involves STING signaling in wound healing in DW. HG-induced STING activation promoted M1 polarization of macrophages and aggravated DW healing disorder.

In conclusion, we demonstrated that STING signaling was involved in the development of DW refractory by activating the pro-inflammatory effects of macrophages. We also demonstrated the existence of STING activation in human DW lesions and the therapeutic effect of gene-edited to silence STING in macrophages in promoting wound healing in diabetic mice, providing a solid basis for clinical translation. We have reason to believe that the targeted inhibition of STING, which induced the polarization of wound macrophages from pro-inflammatory M1 to anti-inflammatory M2, would provide positive and far-reaching beneficial effects on the prevention or treatment of DW (Fig. 8).

## Materials and Methods

### Clinical tissue samples

The clinical tissue samples were obtained from 27 patients, including 9 normal skin tissues from non-diabetic patients (NC, 5 males and 4 females), 9 traumatic wound tissues from non-diabetic patients (NDW, 5 males and 4 females), and 9 wound tissues of diabetic patients (DW, 6 males and 3 females). All patients excluded hypertension, hyperlipidemia, lower extremity vascular occlusion, pregnancy, autoimmune diseases, skin allergies, blood disease, mental disorders, hormone or intravenous drug addiction history. All tissue samples were from the lower extremities. The samples in the NC group were obtained from the discarded full-thickness skin grafts from the donor site of scar surgery. The samples in the NDW and DW groups were obtained from the discarded debridement and excision tissue in the surgical area. All experimental subjects and experimental material collection operations were reviewed and approved by the Ethics Committee of The Affiliated Hospital of Southwest Medical University. All patients involved in tissue samples had been fully informed and voluntarily signed the informed consent for the collection of clinical tissue samples before surgery. And then approved by the Medical Department of The Affiliated Hospital of Southwest Medical University.

### Animal experiment

Wild-type (WT) C57BL/6 J mice (4–5 weeks old) were obtained from Dashuo Biotechnology Co. Ltd (Chengdu, China). STING conventional knockout (STING^−/−^) mice (C57BL/6 N background) were obtained from Saiye Biotechnology Co. Ltd (Suzhou, China). db/db and db/m mice (4–5 weeks old) were obtained from TengXin Biotechnology Co. Ltd (Chongqin, China). All animals were housed in specific-pathogen-free (SPF) rated animal rooms (humidity 50 ± 5%, temperature 23 ± 1 °C), with a day-night cycle of 12 h light and 12 h dark (lights on at 6:00 AM). 25 WT C57BL/6 J mice received a saline injection and fed with a Mouse maintenance diet from Dashuo Biotechnology Co. Ltd (Chengdu, China) as control, while 50 mice (*n* = 50) received Streptozotocin (STZ, Sigma, USA) at a dose of 50 mg/kg (i.p., 0.1 mmol/L sterile sodium citrate buffer) for 5 consecutive days after 12 weeks of a high-fat diet (HFD, 60% fat calories) from Trofi Feed Technology Co. Ltd (Nantong, China) to establish a diabetic model. Mice with fasting blood glucose concentrations over 16.7 mmol/L for at least 10 days were considered diabetic mice (DM). Three mice died, and the modeling success rate was 94%. db/db mice were fed with HFD for 2 weeks, those with continuous blood glucose levels over 16.7 mmol/L were considered diabetic mice. Two weeks after the establishment of a diabetic model. Mice were anesthetized with pentobarbital sodium from Yuanye Biotechnology Co.Ltd (Shanghai, China) at a dose of 50 mg/kg. A 10 × 10 mm^2^ punch wound (the wound defect was layered to the upper layer of the fascia and the carcass was removed) were made on the back skin of the mice using a Mouse skin tissue punch sampler from Yuyan Instruments Co. Ltd (Shanghai, China) [26]. Mice treated with STING inhibitor were given an intraperitoneal injection of 750 nmol C-176 (Selleck, USA) per mouse daily in 200 μl corn oil, marked as DM + C176 [27]. Mice treated with STING agonist were given an intraperitoneal injection of DMXAA (Selleck, USA) solution per mouse daily at a dose of 25 mg/kg, marked as DM + DMXAA [28, 29]. Mice treated with Bone Marrow-Derived Macrophage (BMDM) were given a subcutaneous injection of BMDM, BMDM^DMXAA^ and BMDM^STINGKO^ respectively into db/m and db/db mice at 5, 7, and 12 o’clock in the wound margin at 3 days after trauma (0.2 ml/point, cell concentration: 1*10^6/mL) [30], marked as db/m+BMDM, db/db+BMDM, db/m+BMDM^DMXAA^ and db/db+ BMDM^STINGKO^. All animal feeding and experimental procedures were approved by the Ethics Committee of The Affiliated Hospital of Southwest Medical University.

### Cell culture and treatment

Bone marrow cells were isolated from STING^−/−^ and WT mice and differentiated into BMDM [31, 32]. The cell culture conditions used 1640 medium (Thermo, USA) containing 10% FBS (Thermo, USA) and 100 IUml^−1^ penicillin and 100 μg ml^−1^ streptomycin (Beyotime, China). Mice vascular endothelial cells (MVEs) were obtained from ATCC (USA), and the cell culture conditions were following the standards provided by ATCC. All cells were incubated at the temperature of the incubator (Thermo, USA) was 37 °C and the concentration of CO_2_ was 5%. BMDM from STING^−/−^ and WT mice stimulated by NC or HG (30 mmol/L) were treated with DMXAA at a dose of 75 mg/mL (266 nmo/L) [33] or C-176 at a dose of 5 nmo/L. WT and STING^−/−^ BMDM were cultured under NC and HG conditions. And the supernatants were mixed with fresh DMEM medium (Sigma, USA) at a ratio of 1:1 and added to MVEs for co-culture for 48 h. This macrophage-derived conditioned medium named CM, marked as NC-stimulated WT BMDM (NC-CM), HG-stimulated WT BMDM (HG-CM), NC-stimulated STING^−/−^ BMDM (NC-STING^−/−^-CM), HG-stimulated STING^−/−^ BMDM (HG-STING^−/−^-CM).

### Histologic and immunohistochemical assays

Tissue sections were used for histologic and immunohistochemical assays. Hematoxylin-eosin (HE) staining, Masson, and immunohistochemical staining were performed described as follows. Briefly, paraffin-embedded tissue was cut into 5 μm sections, deparaffinized with xylene, and rehydrated by serial of gradient alcohol (100%, 100%, 95%, 90%, 80%, and 70%, 10 min each) firstly. Then the structure of the tissue cells was observed by HE staining (Solebo, China). In addition, collagen fibers and muscle fibers were displayed by Masson (Solebo, China). Finally, the expression and distribution of specific proteins in tissue cells were further evaluated by immunohistochemical staining. Sections were treated at high temperature and pressure for antigen retrieval, and endogenous peroxidase was eliminated by incubating with 3% H_2_O_2_ for 20 min at room temperature, then blocked with 10% goat serum for 1 h. Sections were incubated with primary antibodies against IL-1β (1:100, #12242, CST, USA), CD31 (1:100, sc-376764, Santa, USA), and CD68 (1:100, sc-20060, Santa, USA) overnight at 4 °C. After washing 3 times with PBST, sections were incubated with anti-mouse HRP reagent (1:1000, A0216, Biyuntian, China) for 1 h at 37 °C and DAB for 5 min at room temperature. Finally, the sections were rinsed with water, counterstained with hematoxylin, dehydrated, and observed under a microscope (Leica, Germany).

### Immunofluorescence

Tissue sections were deparaffinized and rehydrated as mentioned above. For colocalization between CD68 and STING, F4/80 and STING, F4/80 and iNOS, and F4/80 and Arg-1, sections were incubated with primary mouse antibody against CD68 (1:80, sc-20060, Santa, USA) and primary rabbit antibody against STING (1:50, #13647, CST, USA), primary mouse antibody against F4/80 (1:80, sc-365340, Santa, USA) and primary rabbit antibody against STING (1:50), primary mouse antibody against F4/80 (1:80) and primary rabbit antibody against iNOS (1:50, #13120, CST, USA), primary mouse antibody against F4/80 (1:80) and primary rabbit antibody against Arg-1 (1:50, #93668, CST, USA) at 4 °C overnight. Then stained with Cy3-labeled goat anti-mouse immunoglobulin G (IgG) H&L secondary antibody (1:200, A0568, Biyuntian, China) or FITC-labeled goat anti-rabbit IgG H&L secondary antibody (1:200, A0562, Biyuntian, China) for 1 h at room temperature, followed by nuclear DAPI (ab104139, Abcam, USA) staining. Finally, Tissues were observed under a Live Cell Workstation (Leica, Germany).

For immunofluorescence in cells, cells were grown on coverslips in 24-well plates, then fixed with 4% paraformaldehyde for 15 min, permeabilized with 0.5% Triton X-100 at room temperature for 10 min, and blocked by 5% bovine serum albumin (BSA) for 1 h. For angiogenesis, cells were incubated with primary antibody against CD31 (1:100, sc-376764, Santa, USA) and VEGF (1:100, sc-57496, Santa, USA) overnight at 4 °C. Then incubated with Cy3-labeled goat anti-mouse immunoglobulin G (IgG) H&L secondary antibody (1:200) for 1 h at room temperature, followed by nuclear DAPI staining. For nuclear translocation, cells were incubated with primary antibody against NF-κB p65 (1:100, #8242, CST, USA) and IRF-3 (1:100, sc-33641, Santa, USA) overnight at 4 °C. Then incubated with FITC-labeled goat anti-rabbit IgG H&L secondary antibody (1:200) for 1 h at room temperature, followed by nuclear DAPI staining. For colocalization between GM130 and STING, dsDNA and Mitofilin, and F4/80 and iNOS, cells were incubated with primary mouse antibody against GM130 (1:100, sc-55590, Santa, USA) and primary rabbit antibody against STING (1:100), primary mouse antibody against dsDNA (1:100, sc-58749, Santa, USA) and primary rabbit antibody against Mitofilin (1:100, ab93323, Abcam, USA), primary mouse antibody against F4/80 (1:100) and primary rabbit antibody against iNOS (1:100) at 4 °C overnight. Then stained with Cy3-labeled goat anti-mouse immunoglobulin G (IgG) H&L secondary antibody (1:200) or FITC-labeled goat anti-rabbit IgG H&L secondary antibody (1:200) for 1 h at room temperature, followed by nuclear DAPI staining. Finally, cells were mounted on a slide and observed under a confocal microscope (Leica, Germany) and a Live Cell Workstation (Leica, Germany).

### Inflammatory cytokines in cell supernatant

Inflammatory cytokines in this study included IL-1β, IL-6, IL-10, and TNF-α. These cytokines in cell supernatant were detected using ELISA kits from Andygene based on the manufacturer’s instructions.

### Flow cytometry

Cells were collected into 1.5 ml EP tubes, after centrifuging and resuspending cells, added 2 µl of the corresponding fluorescent dye-conjugated primary antibody to each EP tube. These dye-conjugated primary antibodies were: FITC anti-mouse CD206 (Biolegend, USA), PE anti-mouse CD11c (Biolegend, USA), APC/Cyanine7 anti-mouse F4/80 (Biolegend, USA), and PE-Cy™7 Rat Anti-CD11b (Biolegend, USA). Then incubated on ice for 30 min, centrifuged, and resuspended cells. Finally, used a FACSAria II four-laser and thirteen-color high-speed flow cytometry sorting system (BD, USA) to complete the Flow cytometric analysis.

### Scratch wound assay

Migration was measured in connection with the application of a wound healing migration assay. Briefly, MVEs were plated in 6-well plates and grown to confluence. A mechanical scratch was made using a 10 μL sterile pipette tip, crossing the center of the confluent cell monolayer and culminating in a denuded area. The scratched monolayer was rinsed extensively with PBS to remove the non-adherent cells as well as the debris, after which BMDM supernatant and a fresh serum-free medium in equal proportions of 1:1 were added with the culture conditions maintained at 37 °C in a 5% CO_2_ humid atmosphere. The denuded area was identified and imaged with a Live Cell Workstation (Leica, Germany) at 0 and 48 h post-wounding.

### Reactive oxygen species (ROS) detection

The fluorescent probe DCFH-DA was used to detect ROS. We detected ROS using an Active oxygen detection kit from Biyuntian based on the manufacturer’s instructions.

### Reverse transcription-quantitative polymerase chain reaction (RT-qPCR)

Total RNA was isolated by Trizol RNA (Invitrogen, USA). Reverse transcription was performed using the Reverse Transcription Kit (Noble, Beijing, China). Quantitative PCR reactions were performed using SYBR Green (Applied Biosystems). Target genes (primers are shown in Table 1, Huada, China) were amplified by a Real-time fluorescence quantitative PCR instrument (Bio-Rad, USA). GAPDH was used as an internal reference and the mRNA relative expression levels of target genes were calculated by the 2-ΔΔCt method. Primer sequences were as follows (Table 1).

**Table 1:** Primer sequences used in this part of the experiment.

Primer Name	Sequence (5’ -> 3’) mouse
STING (F)	AGCGGAAGTCTCTGCAGTCT
STING (R)	GGAGCCCTGGTAAGATCAAC
cGAS (F)	GCCGAGACGGTGAATAAAGT
cGAS (R)	CATTAGGAGCAGAAATCTTCACA
GAPDH (F)	ACTCCACTCACGGCAAATTCA
GAPDH (R)	CGCTCCTGGAAGATGGTG
IL-1β (F)	ACTGTTTCTAATGCCTTCCC
IL-1β (R)	CCAGTTGGTAACAATGCCATGT
TNF-α (F)	CAGGCGGTGCCTATGTCTC
TNF-α (R)	CGATCACCCCGAAGTTCAGTA
IL-6 (F)	GTTCTCTGGGAAATCGTGGA
IL-6 (R)	TGTACTCCAGGTAGCTATGG
Arg-1 (F)	CATGGGCAACCTGTGTCCTT
Arg-1 (R)	CGATGTCTTTGGCAGATATGCA
iNOS (F)	GCCCTGCTTTGTGCGAAGTG
iNOS (R)	AGCCCTTTGTGCTGGGAGTC

### Western blot analysis

Wound tissues or cells were lysed using RIPA buffer (Biyuntian, China) containing PMSF and phosphatase inhibitor followed by centrifugation at 12,000 r/min for 30 min at 4 °C. Total protein concentration in the supernatant was measured by a BCA Protein Concentration Assay Kit (Biyuntian, China). 35 μg samples were separated by sodium dodecyl sulfate-polyacrylamide gel electrophoresis (SDS-PAGE) and then transferred to polyvinylidene fluoride (PVDF) membranes. Membranes were blocked by 5% BSA at room temperature for 1 h and rinsed 3 times with tris-buffered saline with Tween 20 (TBST). Membranes were incubated with primary antibodies against cGAS (1:1000, ab224144, Abcam, USA), STING (1:1000, #13647, CST, USA), NF-κB p65 (1:1000, #8242, CST, USA), p-NF-κB p65 (1:1000, #3033, CST, USA), IRF3 (1:1000, sc-33641, Santa, USA), p-IRF3(1:1000, #29047, CST, USA), IL-1β (1:1000, #12242, CST, USA), CD68 (1:1000, sc-20060, Santa, USA), iNOS (1:1000, #13120, CST, USA), Arg-1 (1:50, #93668, CST, USA), and β-actin (1:5000, Biyuntian, China) at 4 °C overnight. After washing 3 times with TBST, membranes were incubated with horseradish peroxidase-labeled secondary antibodies (1:4000, A0208, and A0216, Biyuntian, China) for 1 h at room temperature. After rinsing 3 times with TBST, membranes were visualized by electrogenerated chemiluminescence (ECL, Santa, USA) in Bio-Rad Image Analysis System (Bio-Rad, USA) and analyzed by Image-J software.

### Statistical analysis

Image analysis using Image J software by selecting ROI analysis area and other data. Statistical analysis was performed using GraphPad Prism 7.0 and SPSS v22.0 software. Measurement data were expressed as mean ± standard deviation of at least three independent experiments. Unpaired t-test was used to compare two groups of unpaired data obeying normal distribution and homogeneity of variance. One-way analysis of variance (ANOVA) and Tukey’s post hoc test were used for comparing data among multiple groups. Differences were considered significant when *p < 0.05, **P < *0.01*, and ***P < *0.001*.

## Supplementary information

Supplemental Material——WB

## Data Availability

All data needed to evaluate the conclusions in the paper are present in the paper and/or the Supplementary Materials. The data that support the findings of this study are available from the corresponding author upon reasonable request.
