# Supplementary material for: High glucose-induced STING activation inhibits diabetic wound healing through promoting M1 polarization of macrophages
Source: Cell Death Discov. 2023 Apr 26;9:136. doi: 10.1038/s41420-023-01425-x (PMC10133226; doi:10.1038/s41420-023-01425-x)
Supplement: Supplementary file 1 — Supplemental Material——WB [file 41420_2023_1425_MOESM1_ESM.pdf]

Fig-1

(B) CD68 IL-1β Actin

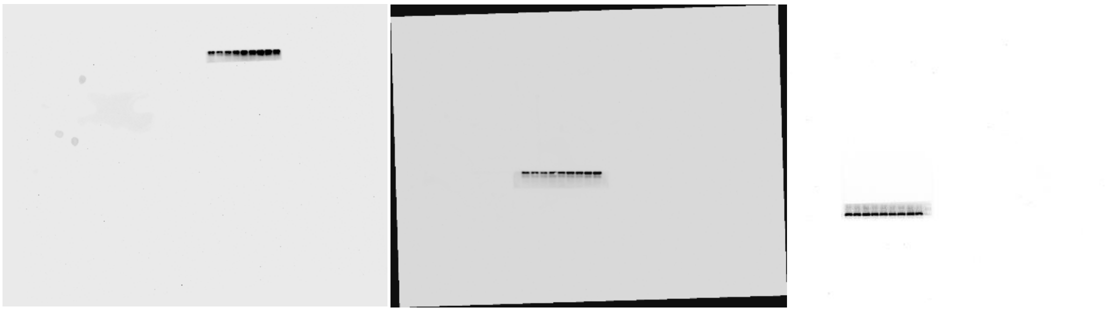

（D） cGAS STING Actin

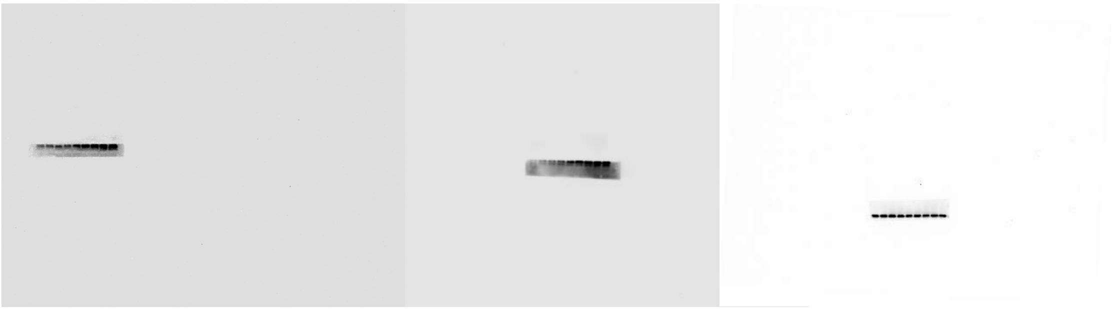

Pp65 P65

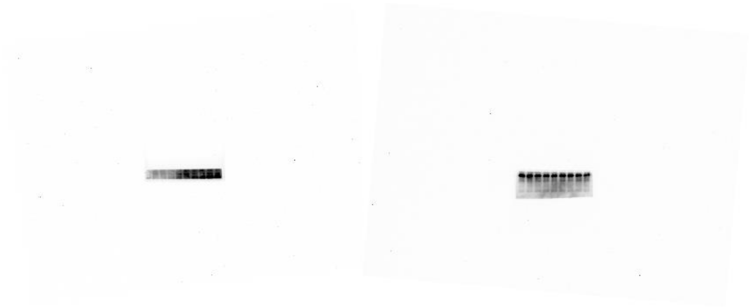

p-IRF3 IRF3

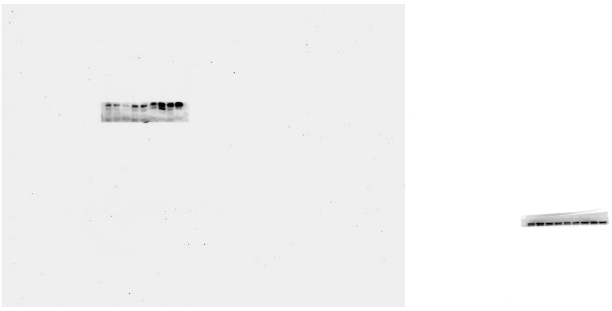

Fig-2

(A) STING Actin

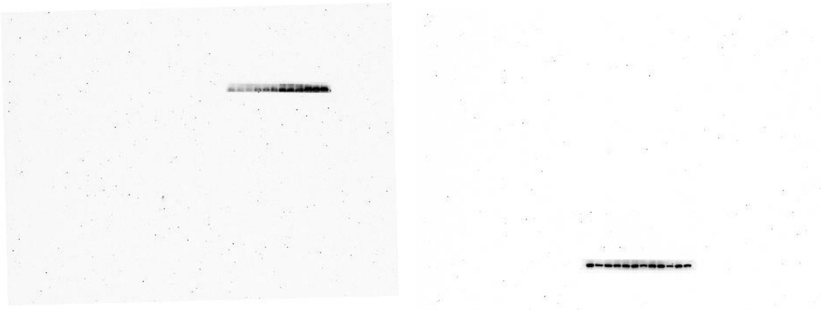

(C) cGAS STING Actin

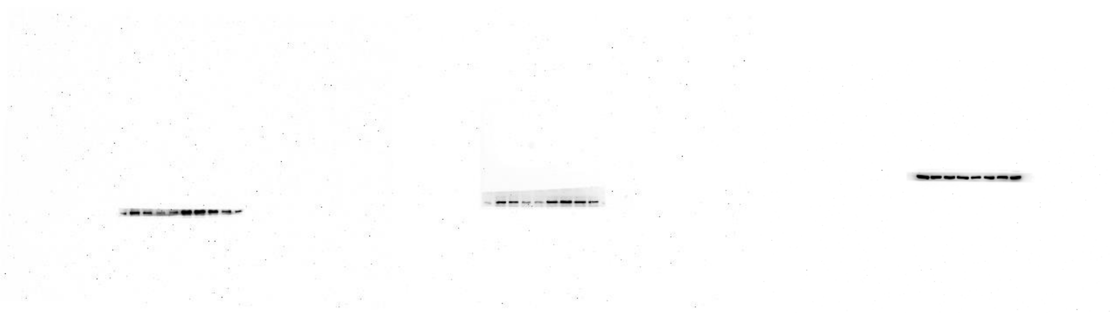

IL-1β

Fig-3

(E) cGAS **STING** **Actin**

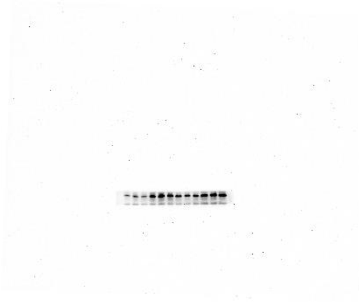

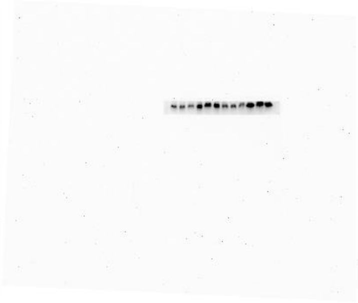

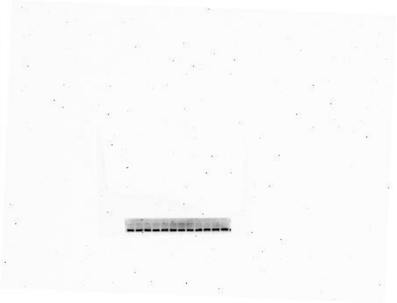

Pp65 **P65**

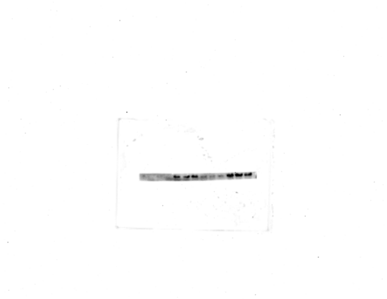

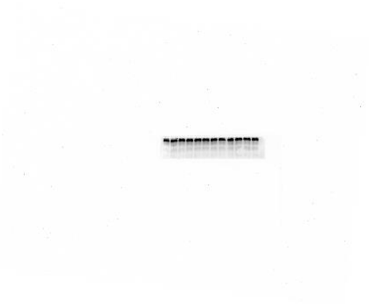

p-IRF3 **IRF3** **IL-1β**

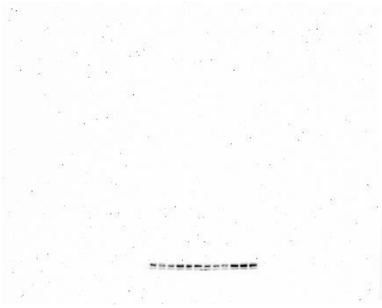

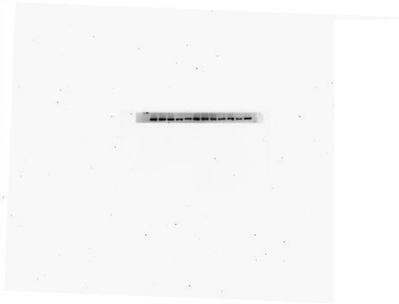

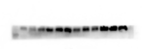

Fig-4

(A) STING **Actin**

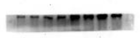

(C) STING **Actin**

(D) Pp65 **P65** **Actin**

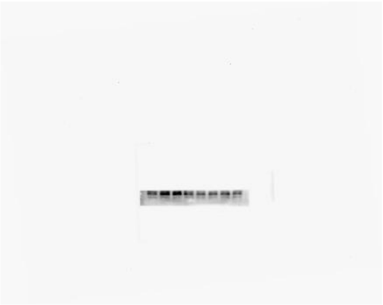

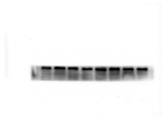

p-IRF3 **IRF3** **IL-1β**

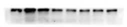

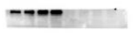

(H) cGAS Actin

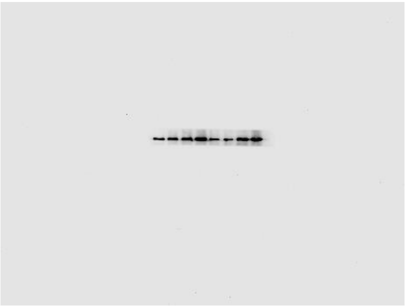

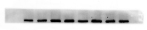

Fig-5

(F) iNOS Arg-1 Actin

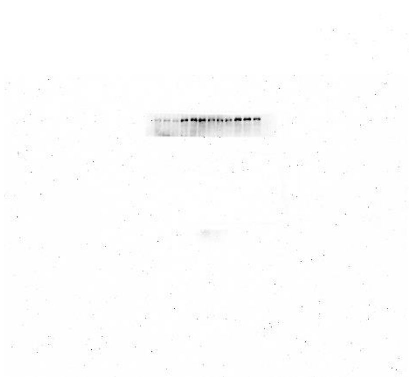

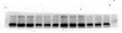

Fig-7

(H) STING

Actin

Pp65

P65

p-IRF3

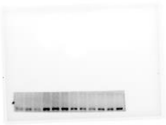

IRF3

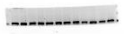

IL-1β

(J) iNOS

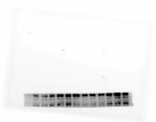

Arg-1

Actin
